# SWI/SNF complex subunit BAF60a represses hepatic ureagenesis through a crosstalk between YB-1 and PGC-1α

**DOI:** 10.1016/j.molmet.2019.12.007

**Published:** 2019-12-20

**Authors:** Wenxiang Zhang, Zhewen Dong, Mengyi Xu, Shiyao Zhang, Chang Liu, Siyu Chen

**Affiliations:** 1 State Key Laboratory of Natural Medicines, China Pharmaceutical University, Nanjing, Jiangsu, 211198, China; 2 School of Life Science and Technology, China Pharmaceutical University, Nanjing, Jiangsu, 211198, China; 3 State key Laboratory of Pharmaceutical Biotechnology, Nanjing University, Nanjing, Jiangsu, 211198, China

**Keywords:** Nutrient signals, BAF60a, YB-1, Urea cycle, Hyperammonemia

## Abstract

**Objective:**

Ureagenesis predominantly occurs in the liver and functions to remove ammonia, and the dysregulation of ureagenesis leads to the development of hyperammonemia. Recent studies have shown that ureagenesis is under the control of nutrient signals, but the mechanism remains elusive. Therefore, intensive investigation of the molecular mechanism underlying ureagenesis will shed some light on the pathology of metabolic diseases related to ammonia imbalance.

**Methods:**

Mice were fasted for 24 h or fed a high-fat diet (HFD) for 16 weeks. For human evaluation, we obtained a public data set including 41 obese patients with and without hepatic steatosis. We analyzed the expression levels of hepatic BAF60a under different nutrient status. The impact of BAF60a on ureagenesis and hyperammonemia was assessed by using gain- and loss-of-function strategies. The molecular chaperons mediating the effects of BAF60a on ureagenesis were validated by molecular biological strategies.

**Results:**

BAF60a was induced in the liver of both fasted and HFD-fed mice and was positively correlated with body mass index in obese patients. Liver-specific overexpression of BAF60a inhibited hepatic ureagenesis, leading to the increase of serum ammonia levels. Mechanistically, BAF60a repressed the transcription of *Cps1*, a rate-limiting enzyme, through interaction with Y-box protein 1 (YB-1) and by switching the chromatin structure of *Cps1* promoter into an inhibitory state. More importantly, in response to different nutrient status, PGC-1α (as a transcriptional coactivator) and YB-1 competitively bound to BAF60a, thus selectively regulating hepatic fatty acid β-oxidation and ureagenesis.

**Conclusion:**

The BAF60a-YB-1 axis represses hepatic ureagenesis, thereby contributing to hyperammonemia under overnutrient status. Therefore, hepatic BAF60a may be a novel therapeutic target for the treatment of overnutrient-induced urea cycle disorders and their associated diseases.

## Highlights

• HFD-feeding increases hepatic BAF60a expression, while inhibits ureagenesis genes.
• BAF60a represses *Cps1* transcription and ureagenesis, causing ammonia accumulation.
• YB-1 binds to BAF60a and mediates the inhibitory effects of BAF60a on ureagenesis.
• BAF60a mediates crosstalk between hepatic ureagenesis and fatty acid oxidation.

## Graphical abstract

**Figure:**
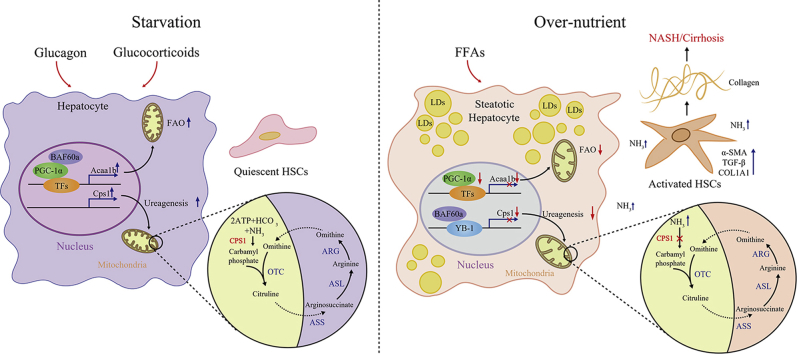

## 1 Introduction

Hepatic ureagenesis plays a predominant role in maintaining nitrogen and ammonia homeostasis in mammals [1]. During prolonged fasting, amino acid (AA) catabolism becomes the main energy supply, accompanied by the generation of ammonia as the end product [2]. Under these conditions, ureagenesis is activated for ammonia detoxification in the liver [1,3]. Given the importance of ammonia clearance, disruption of ureagenesis will lead to the development of hyperammonemia, accompanied by hepatic encephalopathy and liver fibrosis induced by hepatic stellate cell (HSC) activation [4]. Accordingly, it is not surprising that ureagenesis is strictly regulated by external stimuli, especially nutrient signals. In physiological settings, starvation activates the process of ureagenesis, through the increase of hepatic N-acetyglutamamte synthetase/aquaporin-8 activity [5]. In contrast, pathological signals, such as overnutrient states induced by consumption of a high-fat diet (HFD) and high-fat/high-cholesterol (HFHC) diets, impair ureagenesis and cause hyperammonemia [6]. Although these findings are informative, it is still unknown how excess ammonia is produced by the steatotic hepatocytes. Therefore, intensive investigation of the molecular mechanism underlying ureagenesis will shed some light on the pathology of metabolic diseases related to ammonia dysregulation.

Ureagenesis is controlled by five enzymatic reactions that occur sequentially in the mitochondrial matrix and cytoplasm of hepatocytes. These enzymes are carbamoyl phosphate synthetase 1 (CPS1), ornithine transcarbamylase (OTC), argininosuccinate synthetase (ASS), argininosuccinate lyase (ASL), and arginase (ARG) [1]. Among these, CPS1 catalyzes the initial and rate-limiting step wherein ammonia and carbon dioxide combine to form carbamoyl phosphate. CPS1-deficient mice die with overwhelming hyperammonemia [7,8]. Clinically, patients with hyperammonemia caused by genetic CPS1 deficiency often have several pathological syndromes such as encephalopathy, coma, and intellectual disability, suggesting that CPS1 is important for ammonia detoxification [9]. At the molecular level, multiple *cis*-regulatory elements are present on the promoter of *Cps1*. For example, a C/EBPα binding motif is located on the proximal promoter of *Cps1*, which serves as the platform for the competitive binding of Y-box protein 1 (YB-1) and C/EBPα [[10], [11], [12]]. In addition to the traditional transcriptional regulation, recent studies have indicated that *Cps1* expression also undergoes epigenetic regulation. For example, Francesco et al. identified that two CpG islands exist on the *Cps1* promoter, and they are hypermethylated in patients with nonalcoholic steatohepatitis, causing a reduction in *Cps1* transcription [13]. In contrast, fasting- or caloric restriction–induced activation of Sirtuin 3 and 5 deacetylate CPS1 protein increases its activity, leading to the activation of ureagenesis and reduction of ammonia in the liver [2,14]. Although the molecular regulation of ureagenesis has been partially revealed, the comprehensive regulation network integrating nutrient signals and multiple levels of modifications regarding ammonia homeostasis remains elusive.

It has not escaped our notice that various nuclear factors functionally coordinate molecular regulations of metabolic processes in response to nutrient signals. One of the best examples comes from the studies focusing on BAF60a, a subunit of the SWItch/Sucrose NonFermentable (SWI/SNF) complexes [15]. In contrast to other family members, BAF60a responds sensitively to nutrient signals and regulates a series of metabolic pathways. For example, starvation triggers the nuclear translocation of BAF60a onto promoters of genes involved in fatty acid β-oxidation (FAO), while overnutrient signals, such as HFD (60% fat) and Western diet feeding, increase BAF60a expression in the liver [16,17]. As a chromatin remodeling subunit, BAF60a is presented on the proximal promoters of various genes (e.g., *G6Pase* and *Bmal1*) and turns the local chromatin structures into active states [18]. These findings indicate the epigenetic function of BAF60a in regulating gene transcription, highlight the versatile physiological functions of BAF60a, and prompt us to investigate its possible role in the regulation of ureagenesis.

In the present study, we aimed to explore whether BAF60a regulates hepatic ureagenesis and ammonia-induced HSC activation. To answer this question, we used gain- and loss-of-function strategies to manipulate the hepatic BAF60a expression both in vivo and in vitro. Our results demonstrated that BAF60a, as a sensor for malnutritional signals, switches the chromatin structure of *Cps1* (the gene encoding a rate-limiting enzyme in the ureagenesis) promoter into an inhibitory state and represses its transcription. In addition, the peroxisome proliferator-activated receptor-γ coactivator-1α (PGC-1α, as a transcriptional coactivator) and YB-1 competitively bind to BAF60a, thus selectively regulating hepatic FAO and ureagenesis in response to different nutrient states. Our findings strongly suggest that therapeutic intervention targeting BAF60a in the liver may be a promising strategy to treat hyperammonemia and HSC activation-induced fibrosis in patients with nonalcoholic fatty liver disease and nonalcoholic steatohepatitis.

## 2 Materials and methods

### 2.1 Animals

All animal procedures in this investigation conform to the Guide for the Care and Use of Laboratory Animals published by the US National Institutes of Health (NIH publication No. 85-23, revised 1996) and the approved regulations set by the Laboratory Animal Care Committee at China Pharmaceutical University (permit number SYXK-2016-0011). Male C57BL/6 J mice were maintained in a 12-h light–dark cycle and in a temperature- and humidity-controlled environment. For fasting experiments, mice were either fed ad libitum or subjected to 24-h fasting. For HFD-feeding experiments, 10-week-old male C57BL/6 J mice were fed on an HFD (fat content 60%, Research Diets, New Brunswick, NJ, USA) for 16 weeks. For liver-specific overexpression of BAF60a, we transduced a single-stranded adenoviral-associated virus 8 (AAV8) system carrying either BAF60a CDS (accession number NM_031842) or green fluorescent protein (GFP) into mice at a dose of 1 × 10^12^ *vg* through tail-vein injection under the hepatocyte-specific thyroid binding globulin (TBG) promoter. The dose of AAV was chosen based on a previous study showing that this dose functionally manipulates the gene expression in mouse hepatocytes [19,20]. AAV-TGB-BAF60a CDS was generated by homologous recombination. In contrast, to knock down BAF60a expression in liver, AAV8-TBG vector was modified by inserting a human U6 promoter at the Nhe GCGAGC site. To construct AAV8-TBG vectors expressing a BAF60a-specific shRNA, the long oligonucleotide primers were annealed and attached to the viral vector. All AAVs were purified using the AAV Purification Maxiprep Kit (Biomiga, San Diego, CA, USA), and genome copy titers were quantified by real-time quantitative polymerase chain reaction (RT-qPCR) as described previously [21]. Detailed sequences for shRNA oligonucleotide sequences are listed in Table S1. Animals were sacrificed at the indicated time points by cervical dislocation.

### 2.2 Public data sets

Affymetrix Human Gene 1.1 ST microarray data were obtained from the ArrayExpress database, and the data set E-GEOD-48452 was used [22].

### 2.3 Adenovirus, siRNA, and plasmid information

Adenoviruses, including Ad-GFP and Ad-BAF60a, Ad-PGC-1α, Ad-Scra shRNA, and Ad-BAF60a shRNA, and plasmids encoding the Baf60a CDS domain were kindly provided by Prof. Jiandie Lin (Life Sciences Institute, University of Michigan, Ann Arbor, MI, USA) [16]. The plasmid carrying the full-length mouse YB-1 complementary DNA coding sequence and the *Cps1* promoter (−837 to +1) were constructed by Bioworld Company (Nanjing, Jiangsu, China). For YB-1 knockdown, three sets of stealth siRNA were designed, validated, and synthesized by GenePharma (Shanghai, China). To improve gene silencing efficiency, a siRNA cocktail comprising these three sets of siRNA oligonucleotides (an equal molar mixture) was used. The sequences of these siRNA oligonucleotides are listed in Table S1.

### 2.4 Cell culture

Primary hepatocytes (PHs) were isolated from mice using the collagenase IV (Gibco, Grand Island, NY, USA) perfusion method as described previously and cultured in a humidified atmosphere containing 5% CO_2_ at 37 °C [23]. The mouse HSC cell line JS-1 (an immortalized mouse HSC line) was cultured in DMEM supplemented with 10% fetal bovine serum (Sciencell Research, Carlsbad, CA, USA) and 1% antibiotics (penicillin and streptomycin).

### 2.5 RT-qPCR and western blot analyses

Total RNA was isolated using Trizol reagent (Invitrogen, Carlsbad, CA, USA), reverse transcribed, and analyzed by qPCR using SYBR Green (Vazyme, Nanjing, Jiangsu, China) and the LightCycler® 480 System (Roche, Basal, Switzerland). The primers for mouse 36B4 were included for normalization. A complete list of PCR primers is shown in Table S2. For protein expression analysis, liver tissues were homogenized, and the cells were lysed in RIPA buffer. The protein concentration was quantified with a BCA protein quantification kit (Bio-Rad, Hercules, CA, USA). Equal amounts of protein were loaded and separated by 10% sodium dodecyl sulfate–polyacrylamide gel electrophoresis and then transferred onto polyvinylidene difluoride membranes (Millipore, Bedford, MA, USA). The membranes were incubated overnight with appropriate primary antibodies. Bound antibodies were then visualized using horseradish peroxidase–conjugated secondary antibodies. A quantitative analysis was performed by using NIH ImageJ 1.32j software. Western blot results are shown as representative blots from three animals randomly selected from each group. The antibody against BAF60a (Cat. No. 611728; 1:1000 dilution) was purchased from BD Biosciences (San Jose, CA, USA). Antibodies against CPS1 (Cat. No. 18703-1-AP; 1:1000 dilution), OTC (Cat. No. 26470-1-AP; 1:1000 dilution), ASS1 (Cat. No. 16210-1-AP; 1:1000 dilution), ASL (Cat. No. 16645-1-AP; 1:1000 dilution), ARG1 (Cat. No. 16001-1-AP; 1:1000 dilution), NAGS (Cat. No. 21566-1-AP; 1:1000 dilution), α-SMA (Cat. No. 14395-1-AP; 1:1000 dilution), and YB-1 (Cat. No. 20339-1-AP; 1:1000 dilution) were purchased from Proteintech (Chicago, IL, USA). The antibody against PGC-1α (Cat. No. SC-13067; 1:1000 dilution) was purchased from Santa Cruz (Dallas, TX, USA). The antibody against β-actin (Cat. No. BS6007MH; 1:1000 dilution) was purchased from Bioworld Technology (Nanjing, Jiangsu, China).

### 2.6 Alanine tolerance test

To assess the hepatic capacity for urea production in vivo, we conducted an alanine tolerance test as performed previously. Mice were fasted for 16 h and then injected intraperitoneally with 1 mg/kg alanine solution (dissolved in 0.9% saline) [24]. Blood samples were collected at 0, 1, 2, and 4 h after the injection. The serum was subsequently analyzed for urea concentration. The area under the curve (AUC) was calculated and statistically analyzed by Origin8 8.6 (OriginLab, Northampton, MA, USA).

### 2.7 Serological analysis

Blood samples were collected in nonheparinized tubes and centrifuged at 4000 rpm for 10 min at 4 °C. Serum levels of urea and ammonia were determined spectrophotometrically by using commercial kits purchased from BioVision (Mountain View, CA, USA).

### 2.8 Sirius red and hepatic collagen I quantification

Livers were isolated, fixed in 4% paraformaldehyde solution for 24 h in situ, processed for paraffin embedding, and cut into 4-μm transverse sections for Sirius red staining. The sections were photographed with a Nikon fluorescence microscope (200 × magnification, ECLIPSE, Ts2R-FL, Tokyo, Japan). For the collagen I content, a 1% liver homogenate was used for determination of liver collagen I content using an enzyme-linked immunosorbent assay (ELISA) kit from Cloud-clone Corp (Wuhan, Hubei, China).

### 2.9 Co-culture experiments

To mimic the hepatic microenvironment in vitro, we performed the co-culture experiments. Cells were cultured using cell culture inserts (50 μm pore size) to separate both cell populations. JS-1 cells were seeded on the bottom of the plate, while the PHs were plated on the insert. A ratio of PHs/JS-1 of 5:1 was chosen according to a previous study [25]. After 48-h co-culture, JS-1 cells were collected for the analysis of α-SMA expression by Western blot or immunocytochemistry (ICC) analyses.

### 2.10 ICC analysis

JS-1 cells were fixed in ice-cold 4% paraformaldehyde for 30 min, followed by blocking in 5% goat serum for 1 h, and incubation with rabbit polyclonal anti–α-SMA antibody overnight at 4 °C. After repeated washing, the cells were probed with secondary antibodies conjugated to Alexa Fluor 488 anti-rabbit immunoglobulin G (IgG; Cat. No. 4408 S, 1:500 dilution, Cell Signaling Technology, Danvers, MA, USA) for 1 h at room temperature. Nuclei were identified with DAPI. The sections were photographed with a Nikon fluorescence microscope (ECLIPSE, Ts2R-FL, Tokyo, Japan).

### 2.11 Transfection and reporter gene assays

The YB-1 binding site on the *Cps1* promoter was mutated and synthesized by Bioworld (Nanjing, Jiangsu, China). All transient transfections were conducted using Lipofectamine 3000 (Invitrogen, Carlsbad, CA, USA) according to the manufacturer's instructions. For the luciferase reporter assays, 200 ng of reporter plasmids was mixed with 50 ng of expression constructs for YB-1 in the presence or absence of 500 ng BAF60a expression construct. Equal amounts of DNA were used for all transfection combinations by adding the appropriate vector DNA. Relative luciferase activities were determined 48 h following transfection using the Luciferase System (Promega, Madison, WI, USA). The data were representative of at least six independent experiments.

### 2.12 Co-immunoprecipitation (Co-IP) analysis

Liver tissues were homogenized, and the cells were lysed in the IP lysis buffer. After centrifugation, 40 μg of protein lysate was incubated with 20 μL of protein-A/G agarose beads (Roche, Basal, Switzerland) and 1 μg of anti-BAF60a or anti–YB-1 antibody. After overnight incubation, the immune complexes were centrifuged and washed four times with ice-cold IP wash buffer. The immunoprecipitated protein was further analyzed using Western blotting assay.

### 2.13 Chromatin immunoprecipitation (ChIP) assay

ChIP assays were performed in mouse PHs essentially as described previously. Briefly, chromatin lysates were prepared, precleared with protein-A/G agarose beads, and immunoprecipitated with antibodies against BAF60a, YB-1, PGC-1α, and K4-trimethylated histone H3 (H3K4-Me3, Cat. No. ab1012, Abcam, Cambridge, MA, USA), K9-dimethylated histone H3 (H3K9-Me2, Cat. No. ab1220, Abcam, Cambridge, MA, USA), or normal mouse IgG (Cat. No. SC-2025, Santa Cruz, Dallas, TX, USA) in the presence of bovine serum albumin (BSA) and salmon sperm DNA. The beads were extensively washed before reverse cross-linking. The DNA was purified using a PCR purification kit (Qiagen, Valencia, CA, USA) and subsequently analyzed by PCR using primers flanking the proximal binding sites for YB-1 on the mouse *Cps1* promoter, as detailed in Table S2. For the primer sequences flanking the proximal binding sites for BAF60a and PGC-1α on the mouse, the *Acaa1b* promoter was synthesized according to our previous studies.

### 2.14 Oxygen consumption rate assay

The **o**xygen consumption rate was analyzed using a Seahorse XF24 Extracellular Flux Analyzer (Seahorse Bioscience, North Billerica, MA, USA). In brief, 2 × 10^5^ PHs were seeded into each well of a Seahorse XF24 cell culture microplate. On the next day, the cells were transfected with either vector or YB-1 plasmid for 48 h; 1 μM dexamethasone and 10 μM forsklin were added when necessary. Prior to the assay, the medium was changed to DMEM containing 25 mM glucose, 1 mM pyruvate, and 4 mM glutamine, and the cells were equilibrated for 1 h at 37 °C. During measurement, oligomycin (final concentration 1 μM), carbonyl cyanide-p-trifluoromethoxy phenylhydrazone (FCCP, final concentration 1 μM), rotenone, and antimycin (Rote/AA, final concentration 1 μM) were sequentially injected into each well at the indicated time points. Data were derived from Seahorse XF24 Wave software and displayed in pmol/min.

### 2.15 Statistical analysis

Statistical analysis was performed using the Origin 8 software (version 8.6, OriginLab Corporation, USA). Groups of data were presented as the mean ± SD (standard deviation). One-way analysis of variance followed by Fisher's least significant difference *post hoc* test were performed to analyze the data. A *P* value < 0.05 was considered statistically significant.

## 3 Results

### 3.1 Hepatic BAF60a and ureagenesis-related genes are regulated by nutrient signals

**Figure 1:**
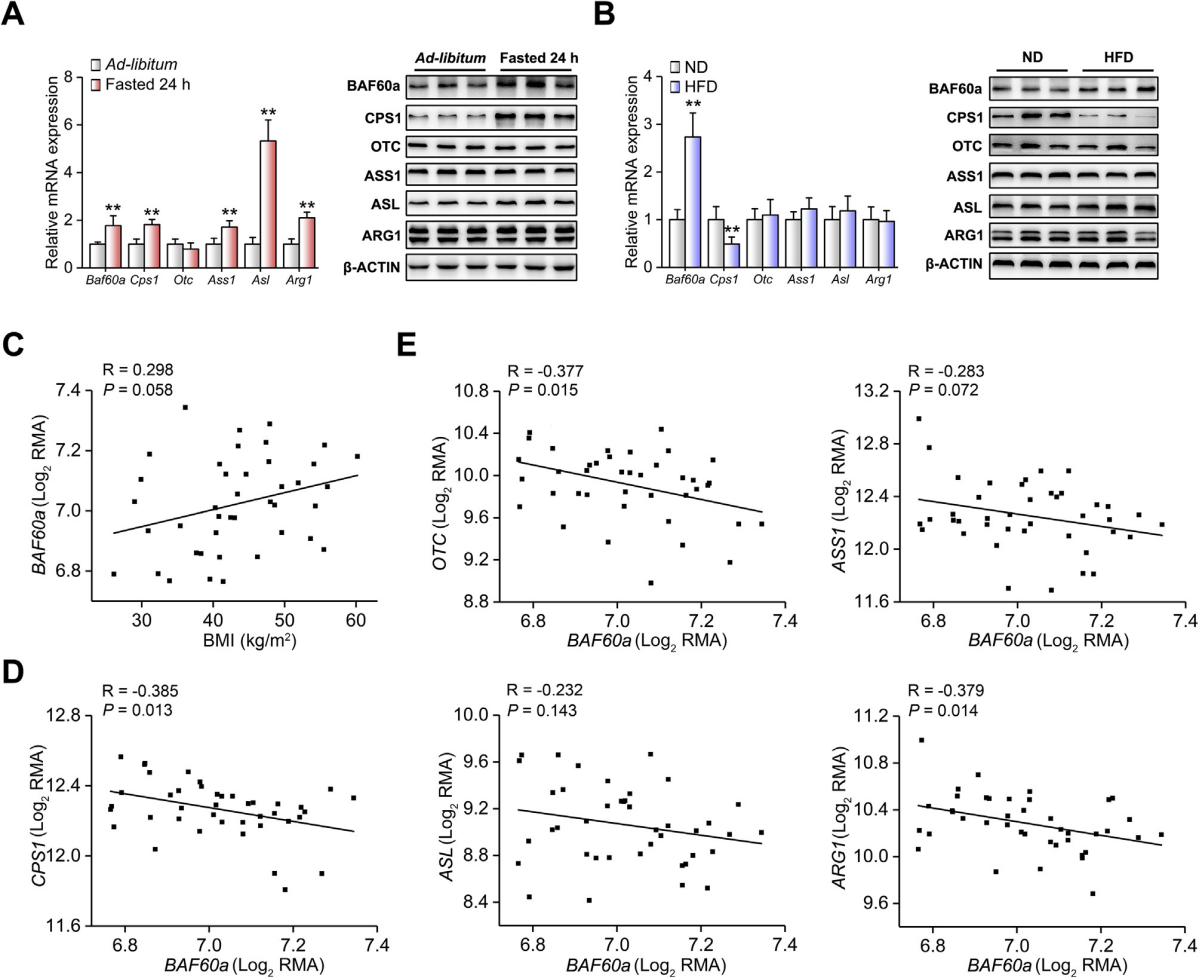
Hepatic BAF60a and ureagenesis-related genes are regulated by nutrient status. (A) Quantitative real-time PCR (RT-qPCR) and Western blot analyses of BAF60a and ureagenesis gene expression in the liver from mice subjected to either 24-h fasting or ad libitum feeding. **P < 0.01 vs. ad libitum group, n = 5. (B) RT-qPCR and Western blot analyses of BAF60a and ureagenesis gene expression in the liver of mice fed with either a normal diet (ND) or an HFD for 16 weeks. **P < 0.01 vs. ND group, n = 7. All values are presented as mean ± SD. (C) Correlation of hepatic BAF60a mRNA levels with BMI in obese patients. (D-E) Correlation of hepatic mRNA levels of BAF60a with CPS1 (D) and other ureagenesis-related genes (E) in obese patients. Data were downloaded from GEO database (E-GEOD-48452) and normalized using Log_2_RMA. N = 41 (healthy obese = 27, steatotic obese = 14).

As is known, fasting induces ureagenesis in the mouse liver [1,3,26]. As expected, the hepatic mRNA and protein expression levels of ureagenesis-associated genes, including CPS1 and ASL, were significantly increased by 24-h fasting. Interestingly, BAF60a expression was also induced in the fasted mouse liver (Figure 1A and Fig. S1A). In coincidence with the in vivo results, these genes were upregulated in mouse PHs treated with dexamethasone and forskolin (DF), an in vitro model mimicking fasting signals [27] (Figs. S1B and S1C). On the contrary, HFD induces obesity and impairs hepatic ureagenesis in mice [6]. We found that while CPS1 expression was inhibited in the liver of HFD-fed mice, BAF60a expression was still increased in response to overnutrient signals (Figure 1B and Fig. S1D). Similar results were observed in mouse PHs treated with a mixture of free fatty acids (FFAs; mixture of oleic acid and palmitic acid in a molar ratio of 1:1; Figs. S1E and S1F). In contrast, the hepatic mRNA expression levels of *Baf60b* and *Baf60c*, two more BAF60 family members, remained unchanged in all the examined settings (Figs. S2A–D).

We next explored the relevance of BAF60a and ureagenesis in humans. We first analyzed the correlation between hepatic mRNA expression of BAF60 family members and body mass index (BMI) and found that the expression of *BAF60a*, but not *BAF60b* or *BAF60c*, was positively correlated with BMI in obese patient cohorts (*R* = 0.298 and *P* = 0.058, Figure 1C and Figs. S2E and S2F). Second, the mRNA levels of *BAF60a* and ureagenesis-related genes were negatively correlated (for *CPS1*, *R* = −0.385 and *P* = 0.013; for *OTC*, *R* = 0.377 and *P* = 0.015; for *ARG1*, *R* = −0.379 and *P* = 0.014) in the liver of obese patients (Figure 1D,E).

Taking these data together, we conclude that BAF60a may serve as a specific component of the BAF60 family linking the nutrient signals to hepatic ureagenesis.

### 3.2 BAF60a inhibits hepatic ureagenesis and triggers HSC activation

**Figure 2:**
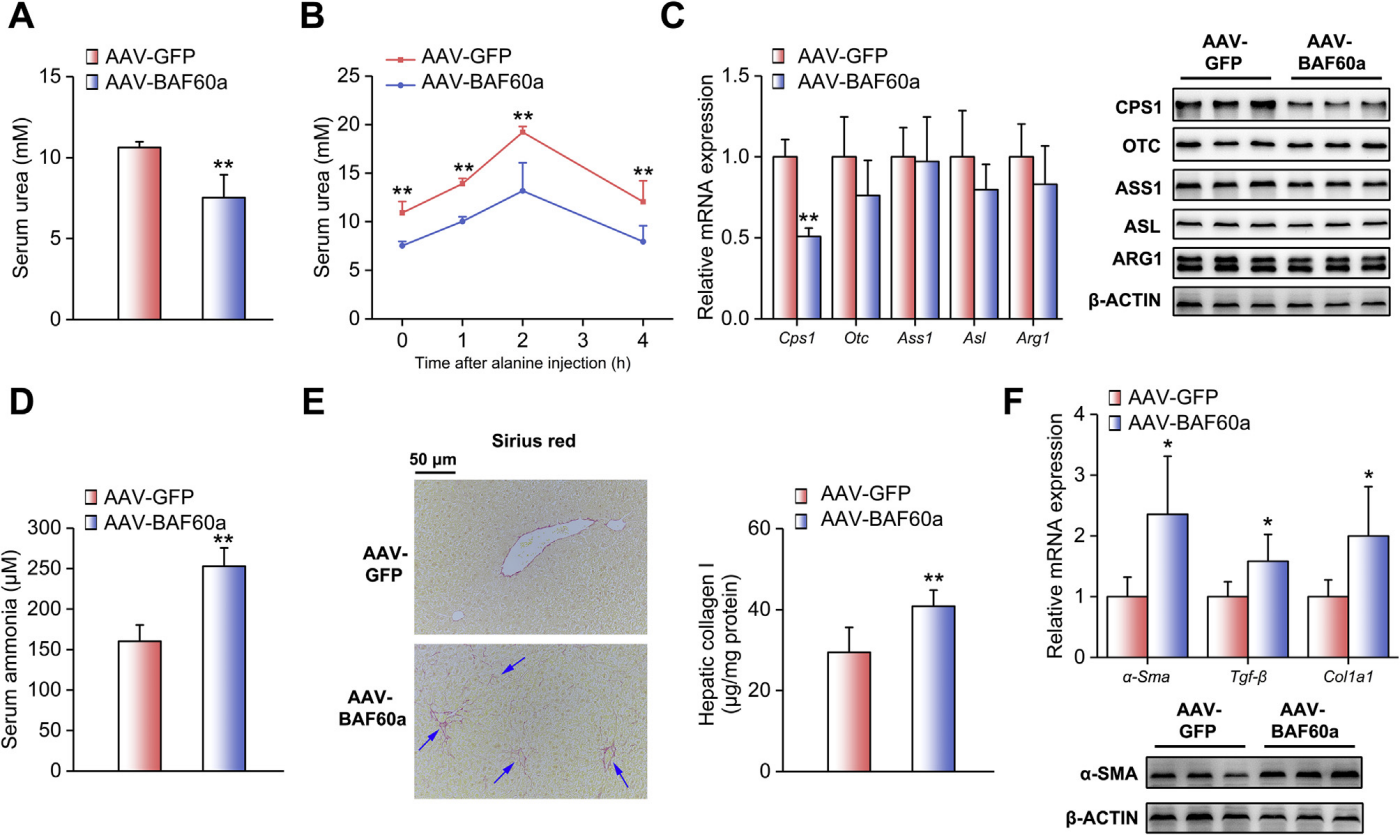
BAF60a inhibits hepatic ureagenesis and triggers HSC activation in vivo. Mice were transduced with the AAV8 system carrying BAF60a CDS at a dose of 1 × 1012 vg through tail-vein injection for 8 weeks. Before being sacrificed, mice were fasted for 24 h n = 5. (A) Serum urea levels. (B) Alanine tolerance tests. (C) RT-qPCR and Western blot analyses of ureagenesis gene expression. (D) Serum ammonia levels. (E) Hepatic collagen I contents detected by Sirius red staining (left) and ELISA kit (right). (F) RT-qPCR analysis mRNA levels of *α-Sma*, *Tgf-β*, and *Col1a1* expression (top) and Western blot analysis of α-SMA protein expression (bottom). *P < 0.05 and **P < 0.01 vs. AAV-GFP group. All values are presented as the mean ± SD.

To examine the liver-specific role of BAF60a in the regulation of ureagenesis and ammonia-induced HSC activation in vivo, we constructed mice with liver-specific BAF60a overexpression by using the AAV8 system carrying a BAF60a CDS domain. The overexpression efficiency and specificity were validated in Figs. S3A and S3B. As shown in Figure 2A, the basal serum levels of urea during starvation were lower in mice with hepatic BAF60a overexpression. These mice also exhibited impaired alanine tolerance (Figure 2B and Fig. S3C). Consistently, the hepatic expression levels of CPS1 were reduced (Figure 2C and Fig. S3E). Of note, given the importance of NAGS in regulating CPS1, we further detected hepatic expression levels of NAGS in mice with hepatic BAF60a overexpression and found that overexpression of BAF60a modestly altered the expression level of NAGS (Figs. S3D and S3E). As reported, reduction of hepatic ureagenesis resulted in a remarkable increase in the serum ammonia levels and the activation of HSCs [4]. Indeed, overexpression of BAF60a increased fasting serum ammonia levels by 57.7% (Figure 2D). Meanwhile, mild portal fibrosis and collagen I deposition were observed in the liver of these mice (Figure 2E). At the molecular level, overexpression of BAF60a increased the mRNA expression levels of *α-Sma*, *Tgf-β*, and collagen synthesis-associated gene *Col1a1*, which are three markers for HSC activation (Figure 2F). The protein expression of α-SMA was also increased (Figure 2F and Fig. S3F).

**Figure 3:**
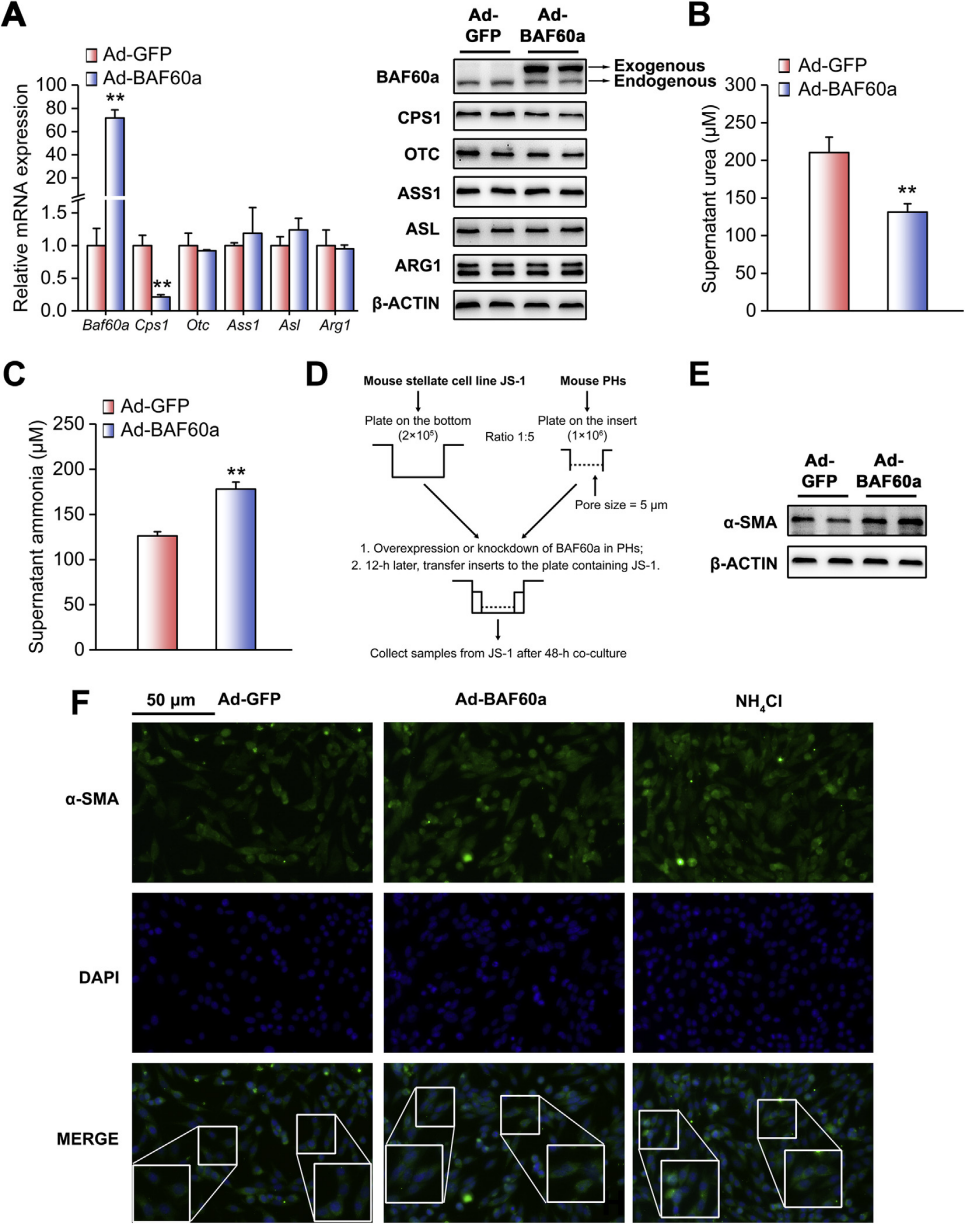
BAF60a inhibits hepatic ureagenesis and triggers HSC activation in vitro. Mouse PHs were infected with adenoviruses encoding GFP (Ad-GFP) or BAF60a (Ad-BAF60a) for 42 h and then treated with the mixture of DF for 6 h. (A) RT-qPCR and Western blot analyses of ureagenesis gene expression. (B) Supernatant urea levels. (C) Supernatant ammonia levels. **P < 0.01 vs. Ad-GFP group, n = 3. (D) A schematic diagram illustrating the co-culture model. Mouse PHs were infected with adenoviruses encoding GFP or BAF60a for 12 h, and then the insert was transferred to the bottom plate containing JS-1 cells. Cells were co-cultured for 48 h, and α-SMA expression was assessed by Western blot (E) and ICC (F) analyses in JS-1 cells. Forty-eight-hour treatment of 200 μM NH_4_Cl was used as a positive control. All values are presented as the mean ± SD.

Consistent with the in vivo results, we found that exogenous expression of BAF60a in DF-treated mouse PHs inhibited CPS1 expression (Figure 3A and Fig. S3G). Functionally, overexpression of BAF60a decreased urea production and increased ammonia concentrations in DF-treated PHs (Figure 3B,C). We next performed a co-culture experiment to mimic the crosstalk between PHs and HSCs in vitro (Figure 3D). Western blot and ICC analyses revealed that BAF60a overexpression in hepatocytes increased the expression levels of α-SMA in JS-1 cells (Figure 3F and Fig. S3H).

Collectively, although BAF60a is induced in the liver of both fasted and HFD-fed mice, these findings provide direct evidence showing that BAF60a is a repressor of hepatic ureagenesis.

### 3.3 Knockdown of BAF60a ameliorates HFD-induced inhibition of ureagenesis and ammonia-induced HSC activation

**Figure 4:**
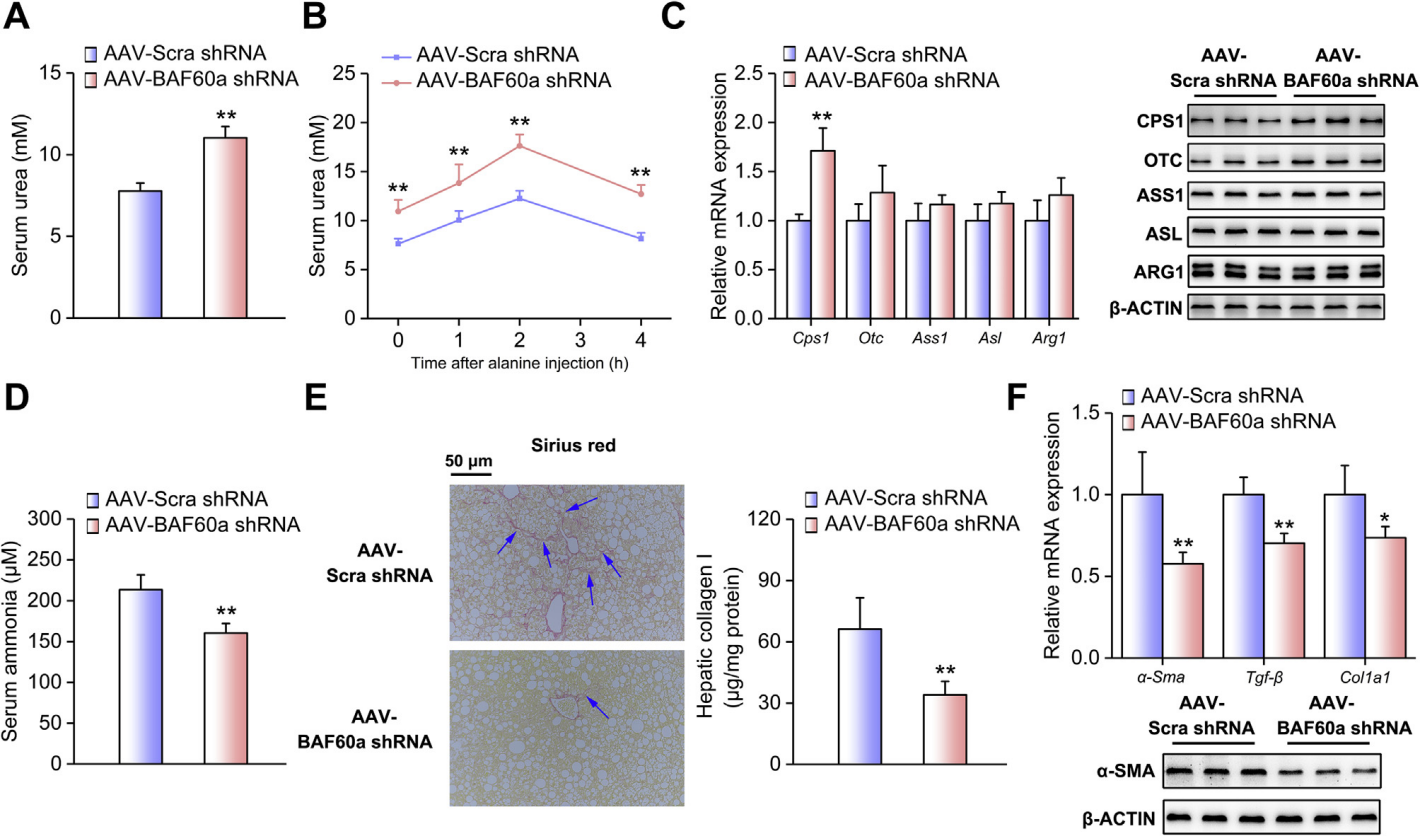
Knockdown of BAF60a ameliorates HFD-induced inhibition of ureagenesis and ammonia-induced HSC activation in vivo. Mice were transduced with the AAV8 system carrying either BAF60a shRNA or Scra shRNA at a dose of 1 × 1012 vg through tail-vein injection, followed by 16-week HFD feeding. n = 5. (A) Serum urea levels. (B) Alanine tolerance tests. (C) RT-qPCR and Western blot analyses of BAF60a and ureagenesis gene expression. (D) Serum ammonia levels. (E) Hepatic collagen I contents detected by Sirius red staining (left) and ELISA kit (right). (F) RT-qPCR analysis of *α-Sma*, *Tgf-β*, and *Col1a1* mRNA expression (top) and Western blot analysis of α-SMA protein expression (bottom). *P < 0.05 and **P < 0.01 vs. AAV-Scra shRNA group. All values are presented as the mean ± SD.

**Figure 5:**
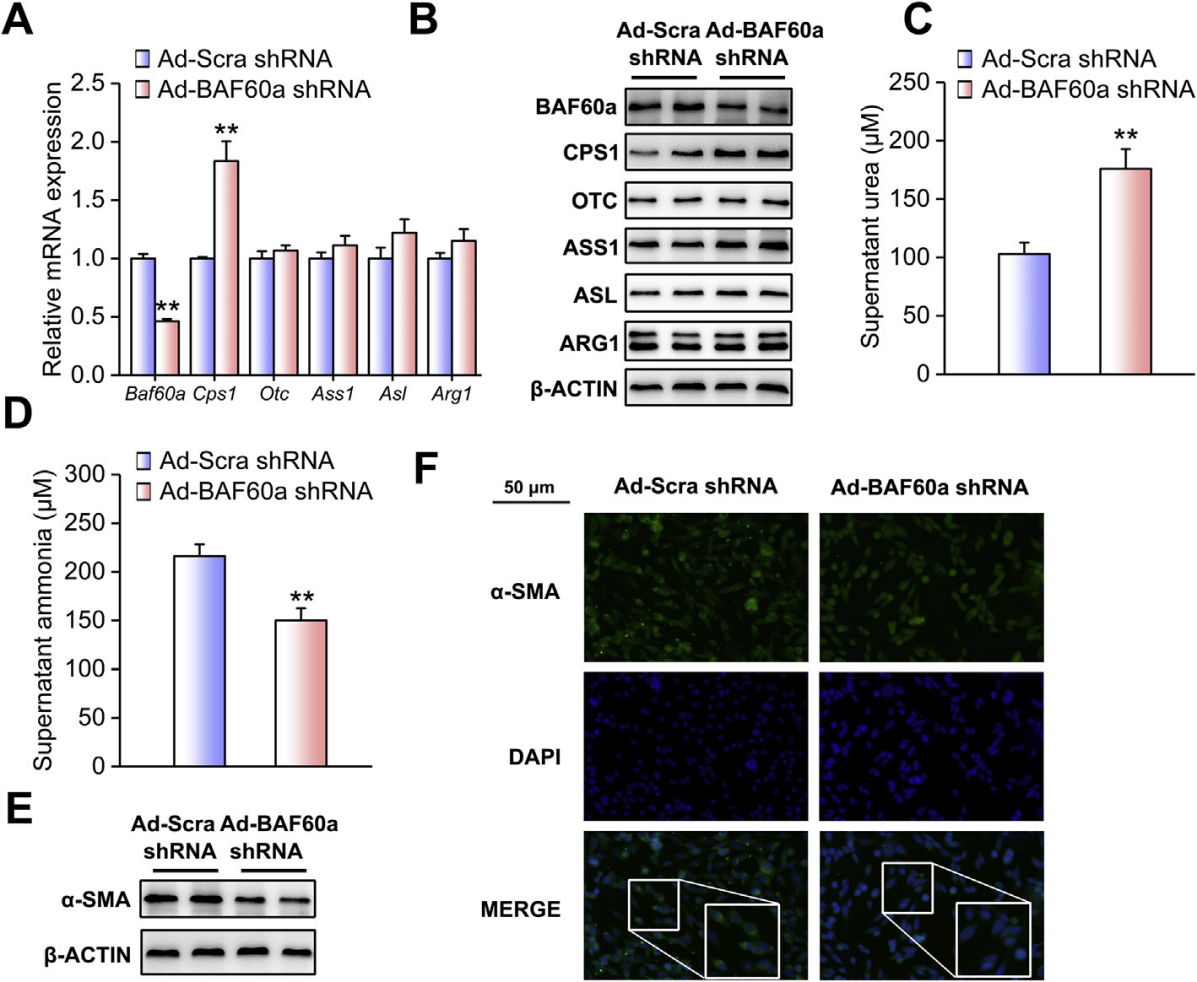
Knockdown of BAF60a ameliorates FFA-induced inhibition of ureagenesis and ammonia-induced HSC activation in vitro. Mouse PHs were infected with adenoviruses encoding Scra shRNA (Ad-Scra shRNA) or BAF60a shRNA (Ad-BAF60a shRNA) for 24 h and then treated with 0.8 mM FFAs for 24 h. (A) RT-qPCR and Western blot analyses of BAF60a and ureagenesis gene expression. (B) Supernatant urea levels. (C) Supernatant ammonia levels. Mouse PHs were infected with the adenovirus carrying either Scra shRNA or BAF60a shRNA for 12 h, and then the insert was transferred to the bottom plate containing JS-1 cells. Cells were co-cultured for 48 h, and α-SMA expression was assessed by Western blot (D) and ICC (E) analyses in JS-1 cells. *P < 0.05 and **P < 0.01 vs. Ad-Scra shRNA group, n = 3. All values are presented as the mean ± SD.

We next adopted a loss-of-function strategy to confirm the inhibitory effects of BAF60a on the ureagenesis process. The knockdown efficiency and specificity were validated in Figs. S4A and S4B. The liver-specific knockdown of BAF60a increased the basal levels of urea in the serum of mice fed an HFD (Figure 4A). Consistently, alanine tolerance was improved in these mice (Figure 4B and Fig. S4C). Moreover, hepatic BAF60a knockdown increased CPS1 expression at both the transcriptional and translational levels (Figure 4C and Fig. S4E), while leaving the NAGS expression remain unchanged (Fig. S4D). As a functional output, ammonia levels were decreased by 24.8% in the serum of these mice (Figure 4D), accompanied by a marked alleviation in hepatic portal fibrosis and collagen I deposition (Figure 4E). In agreement with these results, this treatment reduced the expression levels of *α-Sma*, *Tgf-β*, and *Col1a1* (Figure 4F and Fig. S4F). For the in vitro studies, adenovirus-mediated knockdown of BAF60a in FFA-treated PHs significantly increased CPS1 expression (Figure 5A and Fig. S4G). Functionally, the urea contents were increased (175.9 μM), while ammonia concentrations were decreased by 30.6%, in the supernatant of these cultured cells (Figure 5B,C). Western blot and ICC analyses indicated that the protein contents of α-SMA in JS-1 cells were significantly decreased when BAF60a was knocked down in FFA-treated hepatocytes (Figure 5E,F and Fig. S4H).

### 3.4 BAF60a and YB-1 synergistically inhibit *Cps1* transcription

**Figure 6:**
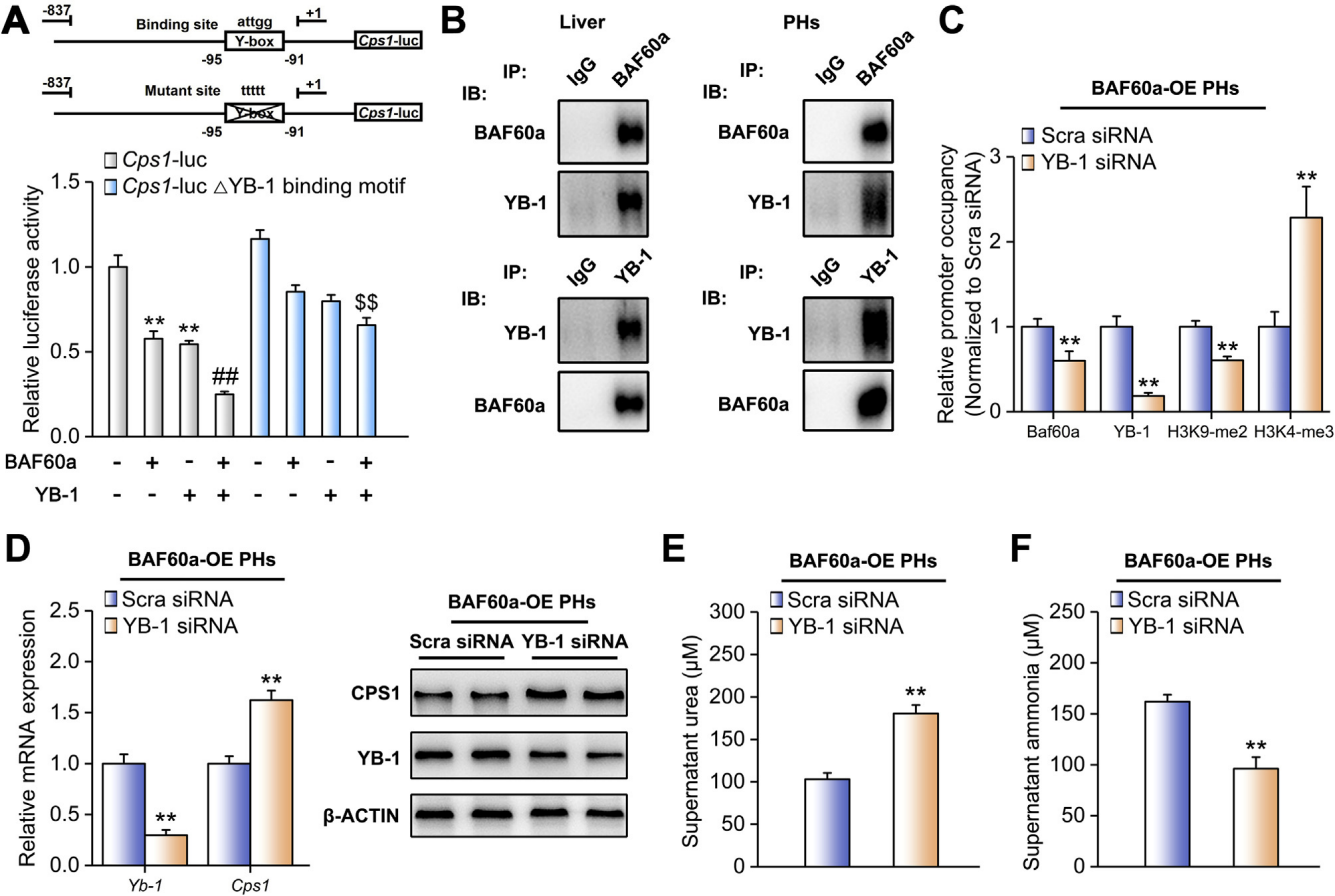
BAF60a and YB-1 synergistically inhibit *Cps1* transcription. (A) Reporter gene assays in mouse PHs transfected with indicated plasmids. **P < 0.01 and ^##^*P* < 0.01 vs. the basal levels, ^$$^*P* < 0.01 vs. co-transfection of BAF60a and YB-1, n = 6. (B) Co-IP assays in the mouse liver homogenates and mouse PH lysates. Immunoblots of precipitated proteins were performed by using indicated antibodies. Mouse PHs were transfected with Scra or YB-1 siRNA for 12 h and then infected by adenoviruses encoding GFP or BAF60a for another 36 h. (C) Chromatin immunoprecipitation (ChIP) assays with indicated antibodies in mouse PHs. The enrichments were quantified by RT-qPCR analysis. OE, overexpression. (D) RT-qPCR and Western blot analyses of YB-1 and CPS1 expression. (E) Supernatant urea levels. (F) Supernatant ammonia levels. **P < 0.01 vs. Scra siRNA group, n = 3. All values are presented as the mean ± SD.

The BAF60 family members were recruited to specific chromatin loci through physical interactions with various transcriptional factors [28]. As CPS1 was the most responsive gene in the above settings, we hypothesized that CPS1 may function as a downstream effector of BAF60a. To identify transcription factors that mediate the repression of *Cps1* transcription by BAF60a, we performed a bioinformatics analysis and found that a classic binding domain for the silencer YB-1 (Y-box) existed on its proximal promoter (−95 to −91 bp). Reporter gene assays demonstrated that BAF60a augmented the inhibitory effects of YB-1 on *Cps1* transcription. However, this synergistic inhibition was partially abolished when the Y-box was mutated (Figure 6A). Furthermore, co-IP assays indicated that endogenous BAF60a and YB-1 physically interacted and formed a complex (Figure 6B). It is known that certain histone modifications, such as H3K9-me2 and H3K4-me3, are associated with chromatin activity [29]. In our study, we found that BAF60a overexpression led to a remarkable increase in H3K9-me2 (repression) levels accompanied by a reduction of H3K4-me3 (activation) levels on the *Cps1* promoter in DF-treated mouse PHs (Fig. S5A). BAF60a knockdown caused the opposite results (Fig. S5B). To confirm that YB-1 is essential for BAF60a-induced CPS1 inhibition, we knocked down YB-1 by a siRNA cocktail in mouse PHs. As shown in Figure 6C, YB-1 knockdown attenuated BAF60a-induced epigenetic inactivation of the *Cps1* promoter. Consequently, YB-1 siRNA increased CPS1 expression in BAF60a-overexpressed PHs (Figure 6D and Fig. S5C). Functionally, urea levels were increased, while the ammonia concentrations declined, in the supernatant of these cultured cells (Figure 6E,F). Taken together, YB-1 is a bona fide chaperon for BAF60a to synergistically inhibit CPS1 expression and ureagenesis.

### 3.5 BAF60a mediates crosstalk between hepatic ureagenesis and FAO pathways

**Figure 7:**
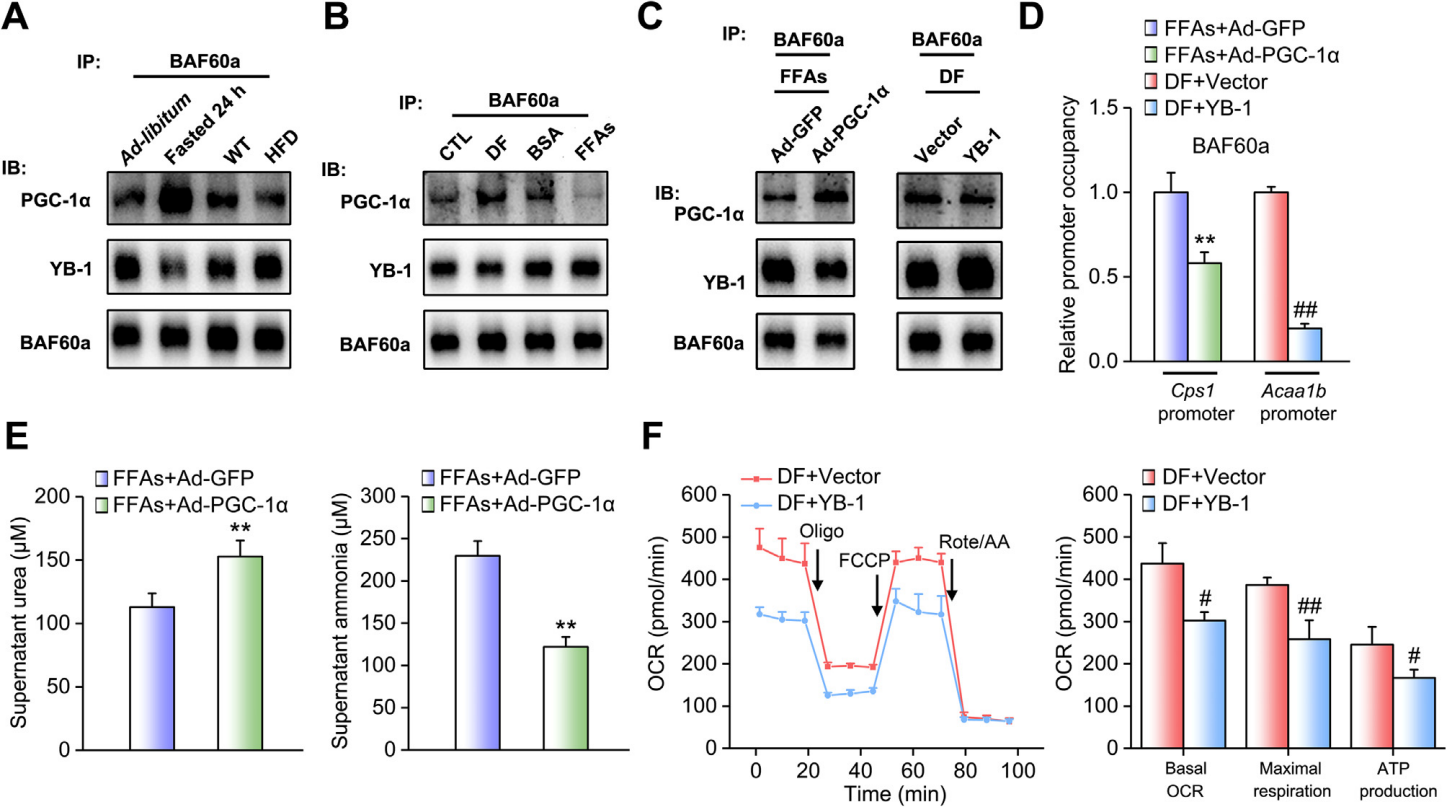
BAF60a mediates crosstalk between hepatic ureagenesis and FAO pathways. (A) Co-IP assays in the liver lysates from mice subjected to 24-h fasting or HFD feeding. Immunoblots of precipitated proteins were performed by using indicated antibodies. n = 3. (B) Co-IP assays in mouse PHs treated with either DF for 6 h or FFAs for 24 h. Immunoblots of precipitated proteins were performed by using indicated antibodies. (C) Left: Co-IP assays in mouse PHs infected with adenoviruses encoding GFP (Ad-GFP) or PGC-1α (Ad-PGC-1α) followed by 24-h FFA stimulation. Right: Co-IP assays in mouse PHs transfected with vector or the YB-1 plasmid followed by 6-h DF stimulation. Immunoblots of precipitated proteins were performed by using indicated antibodies. (D) ChIP assays with indicated antibodies in mouse PHs treated as above. (E) Supernatant urea (left) and ammonia (right) levels in mouse PHs infected with adenoviruses encoding GFP or PGC-1α followed by 24-h FFA stimulation. (F) Oxygen consumption rate (OCR) analysis in mouse PHs transfected with vector or the YB-1 plasmid followed by 6-h DF stimulation. **P < 0.01 vs. FFAs plus Ad-GFP group; ^##^*P* < 0.01 and ^#^*P* < 0.05 vs. DF plus YB-1 group, n = 3. All values are presented as the mean ± SD.

Although hepatic BAF60a expression is induced in both nutrient-deficient (fasting) and overnutrient (HFD-feeding) conditions, ureagenesis is oppositely regulated in these settings. Thus, we ask how BAF60a regulates this pathway in different ways in response to specific nutrient signals. It has been reported that PGC-1α, a well-known transcriptional coactivator, recruits BAF60a to PPARα-binding sites and activates the transcription of peroxisomal and mitochondrial FAO genes (e.g., *Acaa1b*) under fasting status, whereas its function is abolished in the liver of HFD-fed mice [16,30]. Therefore, we proposed that BAF60a may selectively bind to PGC-1α or YB-1 to regulate FAO or the ureagenesis pathway when the nutrient status changes. Indeed, fasting increased PGC-1α expression in the mouse liver, as expected, and therefore increased the binding of BAF60a to PGC-1α and reciprocally reduced the amounts of BAF60a associated with YB-1. HFD feeding led to opposite results (Figure 7A, Figs. S6A and 6B). These data were confirmed in mouse PHs treated with either DF or FFAs (Figure 7B, Figs. S6C and 6D). More importantly, we overexpressed PGC-1α in FFA-treated mouse PHs and found that PGC-1α interfered with the interaction between BAF60a and YB-1, leading to the reduction of BAF60a recruitment to the *Cps1* promoter. As a result, urea production was increased while the ammonia secretion was decreased in the cell supernatant (Figure 7C–E, Figs. S6E and 6F). On the other hand, we overexpressed YB-1 in DF-treated mouse PHs and found YB-1 similarly impaired the interaction between BAF60a and PGC-1α, resulting in the reduction of BAF60a recruitment to the *Acaa1b* promoter, and finally decreased the FAO rate (Figure 7C,D,F, Figs. S6E and 6F).

## 4 Discussion

The urea cycle is a metabolic pathway for the disposal of excess nitrogen, which arises primarily as ammonia [1]. Although nitrogen is essential for growth and life maintenance, excessive ammonia, usually generated from AA degradation and the impairment of the urea cycle, leads to life-threatening conditions, such as liver fibrosis and hepatic encephalopathy [4]. The molecular mechanisms focusing on the urea cycle itself have been intensively studied, but unfortunately, the relationship between nutritional signals and urea homeostasis remains to be elucidated. Very recently, a paper published in *Hepatology* innovatively revealed that hyperammonemia develops in an nonalcoholic fatty liver disease rodent model [6]. However, how overnutrient signals promote ammonia production in the liver is still an important concern for the better understanding of the pathogenesis of metabolic diseases related to ammonia dysregulation. Given the versatile functions of BAF60a, a nutrient-responsive subunit of SWI/SNF complexes, in the regulation of multiple metabolic processes, we carried out the present study to investigate the role of BAF60a in maintaining urea dynamic balance, especially at the epigenetic level.

Our findings indicated that BAF60a sensitively responded to the external nutrient signals and was induced in the liver of both fasted and HFD-fed mice. Consistently, the hepatic mRNA expression of *BAF60a* was positively correlated with the BMI in the obese patients (Figure 1). Previous studies also demonstrated that this subunit was elevated in the liver of mice fed a Western diet while it was suppressed in the vascular smooth muscle cell layer of aorta from HFD-fed rats [17,29]. The robustness of Baf60a expression in response to malnutrition conditions, and the difference of its regulation in various metabolic tissues/organs, suggests that in contrast to other BAF60 family members, BAF60a is a unique molecule that relays nutritional signals to multiple pathophysiological events at hierarchical levels. Future studies are needed to elucidate how these signals regulate the expression of BAF60a, which is localized in the nuclei, and how the specificity of BAF60a expression in organs is achieved.

As an important enzyme involved in ureagenesis, CPS1 plays a critical role in ammonia detoxification in the first step of the urea cycle [1]. Therefore, it is not surprising that regulation of CPS1 could occur at multiple levels, including transcriptional, translational, and posttranslational modifications, constituting a complicated network for the homeostasis of ureagenesis and ammonia production [2,12]. Previous studies reported that fasting-induced SIRT5 interacts with CPS1 and causes CPS1 deacetylation, revealing a critical regulation mechanism of CPS1 at the posttranslational level [28,31]. We appreciate these important findings, which provided strong evidence for the elucidation of the SIRT5-CPS1 axis. However, these studies focused only on the posttranslational modification of CPS1, masking other possible transcriptional and translational modifications of CPS1 expression. In our study, we found a significant increase in CPS1 expression at both the transcriptional and translational levels in the liver of fasted mice, which differed from previous studies that showed that CPS1 expression remained unchanged in this setting. This discrepancy may be caused by the different time points of sample collection. It should be noted that CPS1 is under the control of the circadian clock, leading to a robust diurnal rhythmicity of its expression. Considering its oscillation, we sacrificed mice and collected all of the samples at ZT12, when the mRNA expression levels of CPS1 consistently reach a peak [32,33]. We believe that selecting such a time window would help to show the real changes of CPS1 expression induced by nutritional signals, excluding the influence of circadian clock. In contrast, since previous studies did not specifically emphasize the sacrifice time, it is possible that the unaltered CPS1 expression they observed is actually the combinational outcomes of clock and fasting regulations. In the HFD setting, it has been shown that a 10-week HFD or 16-week HFHC feeding caused a constitutive reduction in the CPS1 expression at both transcriptional and translational levels in the liver of mice [6]. In accordance with these findings, we found that the hepatic CPS1 protein was repressed in the liver of mice fed a HFD for 16 weeks, indicating that reduction of CPS1 expression is a hallmark of impaired ureagenesis in obese mice. Taken together, our findings add a missing piece to the puzzle of CPS regulation, especially the transcriptional regulation network in response to various nutritional signals.

Gain- and loss-of function studies indicated that BAF60a inhibited hepatic ureagenesis, predominantly through suppression of *Cps1* transcription. As mentioned above, CPS1, the enzyme catalyzing the first and rate-limiting step of the urea cycle, is finely regulated through various mechanisms. Analysis of the *Cps1* promoter revealed that several *cis*-regulatory elements, including the C/EBPα binding motif, glucocorticoid receptor element, and Y-box motif, are required for its promoter activity [10,11]. In addition to these traditional transcriptional regulations, epigenetic modification is also involved to regulate the activities of both *Cps1* promoter (hypermethylation of CpG islands) and CPS1 protein (deacetylation) [2,13,14]. In the present study, we found that BAF60a specifically blocked the first step of the urea cycle and repressed *Cps1* expression at the transcriptional level, thus forming a rapid responsive regulation (Figure 2, Figure 3, Figure 4, Figure 5, Figure 6). Such a prompt regulation guarantees that the urea cycle is sensitive to nutrient signals and could be timely triggered to remove the excessive ammonia. On the other hand, BAF60a, as a subunit of the SWI/SNF complex, regulates *Cps1* transcription by exerting its per se function in the chromatin remodeling. We demonstrated that BAF60a switched the chromatin structure of the *Cps1* proximal promoter from an active state to an inhibitory state under the HFD-fed condition, as evidenced by the increased accumulation of H3K9-me2 (repression) and the decreased contents of H3K4-me3 (activation) around its YB-1 binding site (Figs. S5A and S5B). Notably, BAF60a also similarly alters the chromatin structures around the ROER motif of the clock gene *Bmal1* promoter to activate *Bmal1* transcription in both hepatocytes and vascular smooth muscle cells [18,29]. All these results suggest that BAF60a may regulate its downstream gene expression through a common mechanism, namely, altering the local chromatin structures around potential binding sites of transcriptional factors’ promoters. However, in response to different pathophysiological conditions, BAF60a selectively coactivates different transcriptional factors, leading to the specificity of downstream physiological outputs, thus forming an energy-saving mode to increase the regulatory efficiency in the gene transcription.

The BAF60 family members are recruited to specific chromatin loci through physical interactions with various transcriptional factors [28]. Therefore, we tried to identify the transcriptional factor that mediates the repressive effect of BAF60a on *Cps1* transcription. We found that YB-1 was a bona fide chaperon for BAF60a to synergistically inhibit CPS1 expression and ureagenesis. YB-1 is a transcriptional factor recognizing the *cis*-regulatory element, defined as Y-box (a reverse CCAAT box or ATTGG) [34]. As Y-box is presented in the promoter of many genes, including multidrug-resistant 1, epidermal growth factor receptor, DNA polymerase, and thymidine kinase, YB-1 promotes tumor cell proliferation and is considered as a tumor marker of human hepatocellular carcinoma [35,36]. In contrast, YB-1 also functions as a repressor for various physiological processes. For example, YB-1 suppresses collagen formation and liver fibrosis [37]. More importantly, YB-1 is recruited to the *Cps1* promoter in both the fetal and CCl_4_-injured adult mouse liver and inhibits the C/EBPα-induced transcription of *Cps1* promoter via the Y-box [12]. Similarly, in our study, we found that YB-1 mediated the effects of BAF60a in repressing the *Cps1* transcription and altered the chromatin structure of the *Cps1* promoter. Our findings extend the current understanding of the pathophysiological function of YB-1 in the liver system. In addition, we noticed that YB-1 was quite stable in normal adult liver under physiological conditions (e.g., fasting) but was activated in the steatotic liver under pathological conditions (e.g., HFD feeding). These results indicated that compared with YB-1, BAF60a serves as a major effector responsive for physiological nutrient changes, and the BAF60a/YB-1 complex synergistically functions to produce the inhibitory effects on the metabolic processes, such as ureagenesis. Nevertheless, YB-1 participates in a wide variety of DNA/RNA-dependent events, including DNA reparation, pre-mRNA transcription and splicing, mRNA packaging, and regulation of mRNA stability and translation [[38], [39], [40]]. Therefore, it is of particular interest to explore whether these events are also involved in BAF60a-YB-1 complex-induced repression of the CPS1 expression.

Although hepatic BAF60a expression is induced in both nutrient-deficient (fasting) and overnutrient (HFD-feeding) conditions, ureagenesis is oppositely regulated in these settings. It is important to investigate how BAF60a differently regulates this pathway in response to specific nutrient signals. In our study, we found that PGC-1α competed with YB-1 to recruit BAF60a to PPARα-binding sites and activated the transcription of peroxisomal and mitochondrial FAO genes (e.g., *Acaa1b*) under fasting status. Only when its expression and function are abolished in the liver of HFD-fed mice can YB-1 play a dominant role and recruit BAF60a to the Y-box region of *Cps1* promoter. Our findings established an important crosstalk between the FAO and urea cycle, and BAF60a functions as an integration node for these pathways. Our data demonstrated that the relative abundance of PGC-1α and YB-1 under different nutrient states determines the functions of BAF60a in activating FAO or inhibiting ureagenesis. Under a fasting condition, the BAF60a-PGC-1α complex elevates FAO to maintain the energy supply. In this case, AA degradation is also accelerated so that excessive ammonia is produced and needs to be cleared out promptly. To meet this demand, the formation of BAF60a-PGC-1α complex essentially retards the interaction of BAF60a-YB-1 complex, thus releasing the inhibitory function of BAF60a on CPS1 and activating ureagenesis. In contrast, under the energy-redundant condition (e.g., HFD-feeding), PGC-1α expression is reduced, while hepatic YB-1 and BAF60a expression levels are upregulated. Increased YB-1 competitively binds with BAF60a and decreases the interaction of the BAF60a-PGC-1α complex, thus triggering the inhibition of ureagenesis and FAO. Collectively, under different nutrient statuses, the net output of reciprocal competition between BAF60a-PGC-1α and BAF60a-YB-1 complexes finally determines the on-off switches of ureagenesis and FAO processes.

In conclusion, we elucidated here a BAF60a-orchestrated upstream regulatory mechanism in the urea cycle and provided a convincing explanation for the mechanism through which steatotic hepatocytes produced ammonia via induction of the formation of the BAF60a-YB-1 complex. The attendant crosstalk between ureagenesis and FAO processes protects against excessive accumulation in hepatic ammonia, which otherwise leads to HSC activation in response to overnutrient signals. Therefore, therapeutic intervention targeting BAF60a in the liver may be a promising strategy to treat hyperammonemia and HSC activation-induced fibrosis in patients with nonalcoholic fatty liver disease and nonalcoholic steatohepatitis.

## Author contributions

WXZ, CL, and SYC conceived and designed the research. WXZ and ZWD performed most of the experiments. MYX isolated the PHs from mice. SYZ assisted with all the adenoviral-associated virus constructs. WXZ, MYX, and SYZ performed data analysis and statistical analysis. WXZ, CL, and SYC wrote the paper. All authors reviewed and edited the manuscript and approved the final version of the manuscript.

## Conflict of interest

The authors declare that they have no conflict of interest.
