# Supplementary material for: SWI/SNF complex subunit BAF60a represses hepatic ureagenesis through a crosstalk between YB-1 and PGC-1α
Source: Mol Metab. 2019 Dec 20;32:85–96. doi: 10.1016/j.molmet.2019.12.007 (PMC6953711; doi:10.1016/j.molmet.2019.12.007)
Supplement: Multimedia component 1 [file mmc1.doc]

**SUPPLEMENTARY FIGURES**

**
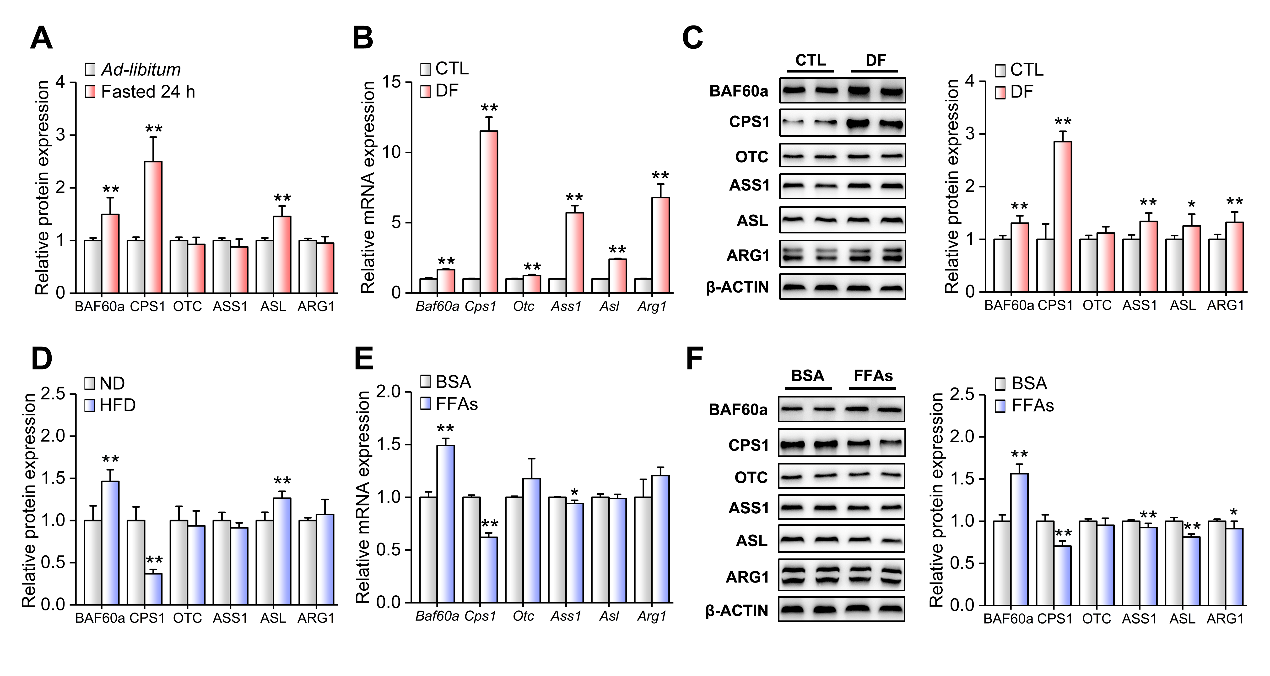
**

**Figure S1. Hepatic BAF60a and ureagenesis-related genes are regulated by nutrient status.** (A) Quantitative analysis of BAF60a and ureagenesis gene protein expression in Figure 1A. ^**^*P* < 0.01 vs. ad libitum group, n = 5. (B) RT-qPCR and (C) Western blot analyses of BAF60a and ureagenesis gene expression in mouse PHs treated with DF for 6 h. ^*^*P* < 0.05 and ^**^*P* < 0.01 vs. CTL group, n = 3. (D) Quantitative analysis of BAF60a and ureagenesis gene protein expression in Figure 1B. ^**^*P* < 0.01 vs. ND group, n = 5. (E) RT-qPCR and (F) Western blot analyses of BAF60a and ureagenesis gene expression in mouse PHs treated with 0.8 mM FFAs for 24 h. ^*^*P* < 0.05 and ^**^*P* < 0.01 vs. BSA group, n = 3. All values are presented as the mean ± SD.


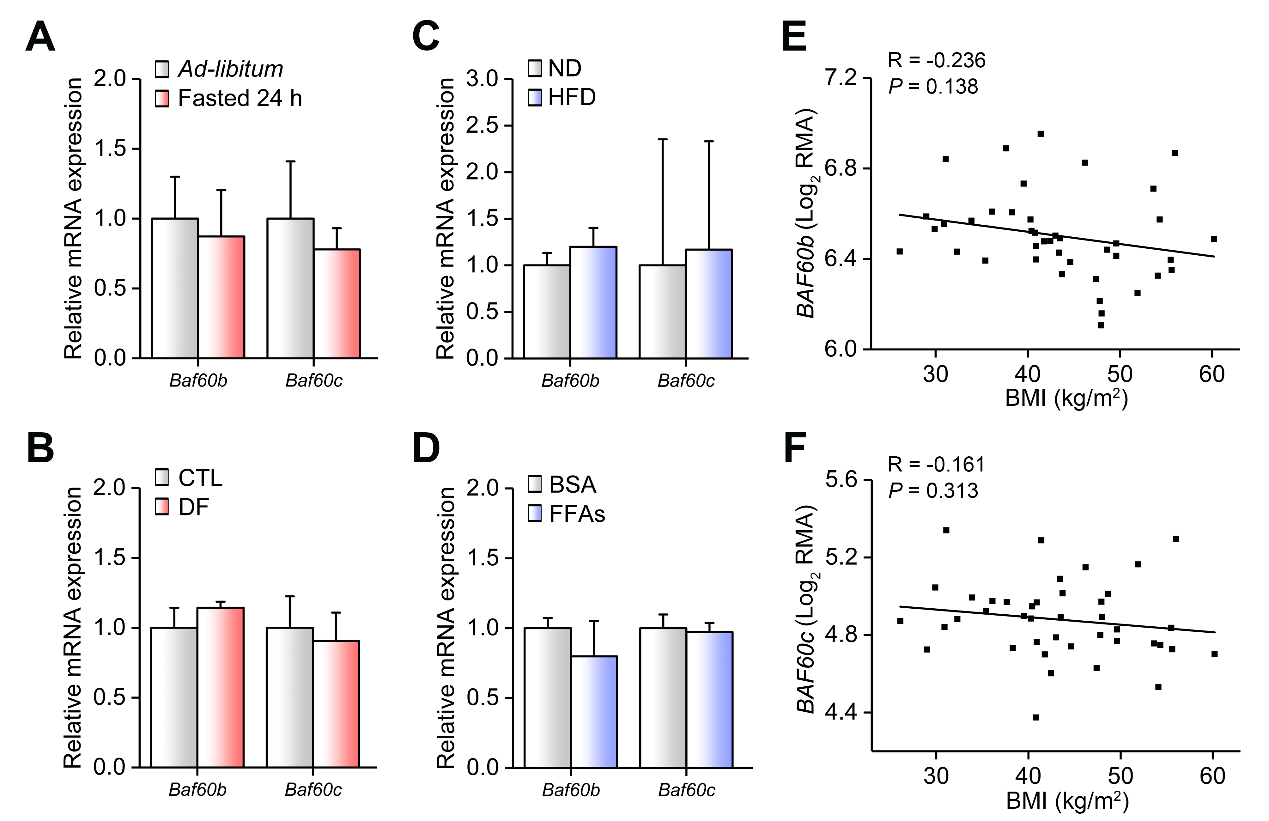


**Figure S2. Hepatic BAF0b and BAF60c are not controlled by nutrient status.** (A) RT-qPCR analysis of *Baf60b* and *Baf60c* mRNA expression in the liver from mice subjected to 24-h fasting. n = 5. (B) RT-qPCR analysis of *Baf60b* and *Baf60c* mRNA expression in mouse PHs treated with DF for 6 h. n = 3. (C) RT-qPCR analysis of *Baf60b* and *Baf60c* mRNA expression in the liver from mice fed with ND or HFD for 16 weeks. N = 7. (D) RT-qPCR analysis of *Baf60b* and *Baf60c* mRNA expression in mouse PHs treated with FFAs for 24 h. n = 3. All values are presented as the mean ± SD. (E-F) Correlation of hepatic *BAF60b* (E) and *BAF60c* (F) mRNA levels with BMI in obese patients. Data were downloaded from GEO database (E-GEOD-48452) and normalized using Log_2_RMA. n = 41 (healthy obese = 27, steatosis = 14).


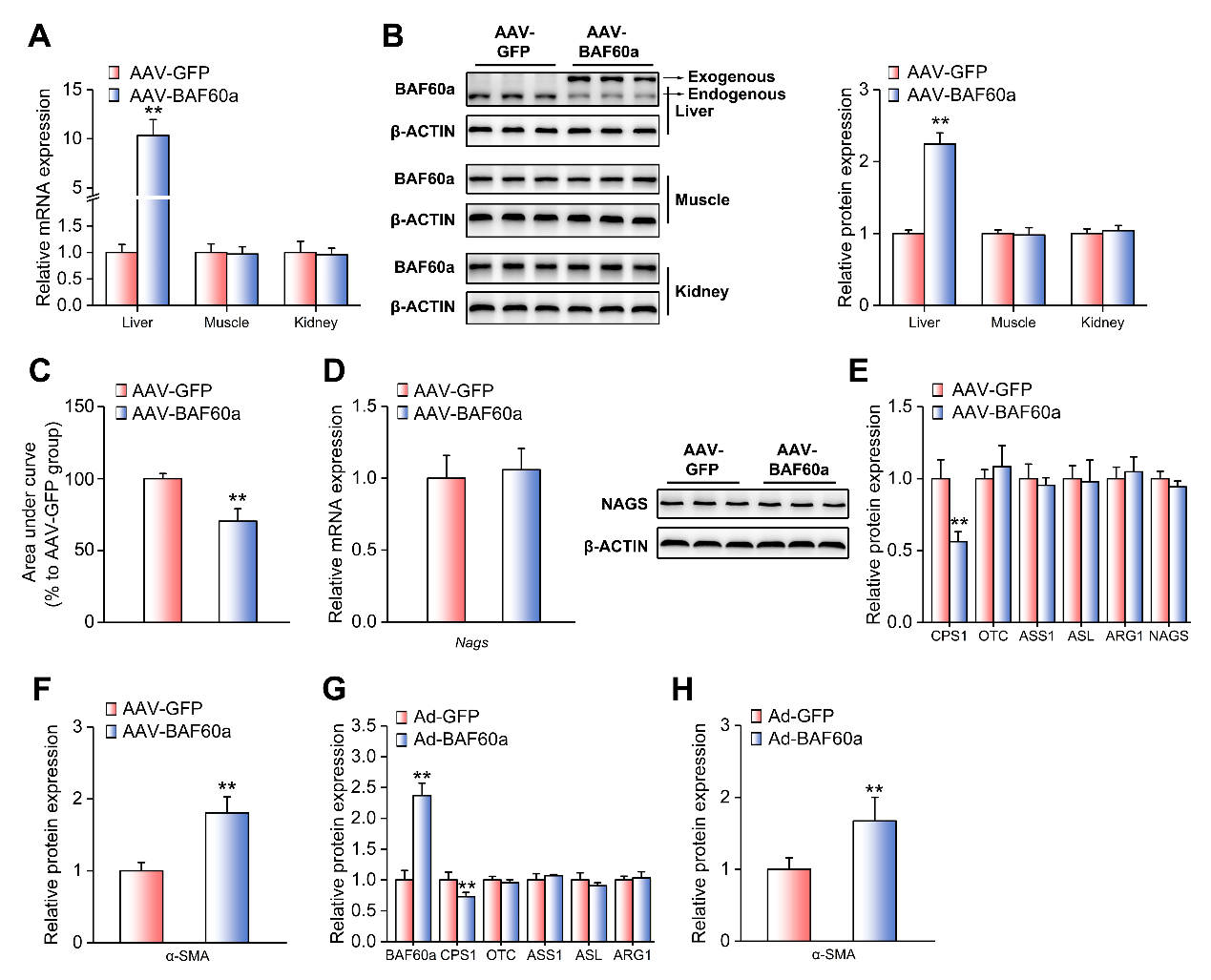


**Figure S3. BAF60a inhibits hepatic ureagenesis and triggers HSC activation*.*** (A) RT-qPCR and (B) Western blot analyses of BAF60a expression in the liver, muscle, and kidney. (C) AUC calculations for alanine tolerance tests. (D) RT-qPCR and Western blot analyses of NAGS expression. (E) Quantitative analysis of BAF60a and ureagenesis gene protein expression in Figure 2C and Figure S3D. (F) Quantitative analysis of α-SMA protein expression in Figure 2F. ^**^*P* < 0.01 vs. AAV-GFP group. n = 5. All values are presented as the mean ± SD. (G) Quantitative analysis of BAF60a and ureagenesis gene protein expression in Figure 3A. (H) Quantitative analysis of α-SMA protein expression in Figure 3E. ^*^*P* < 0.05 and ^**^*P* < 0.01 vs. Ad-GFP group. n = 3. All values are presented as the mean ± SD.


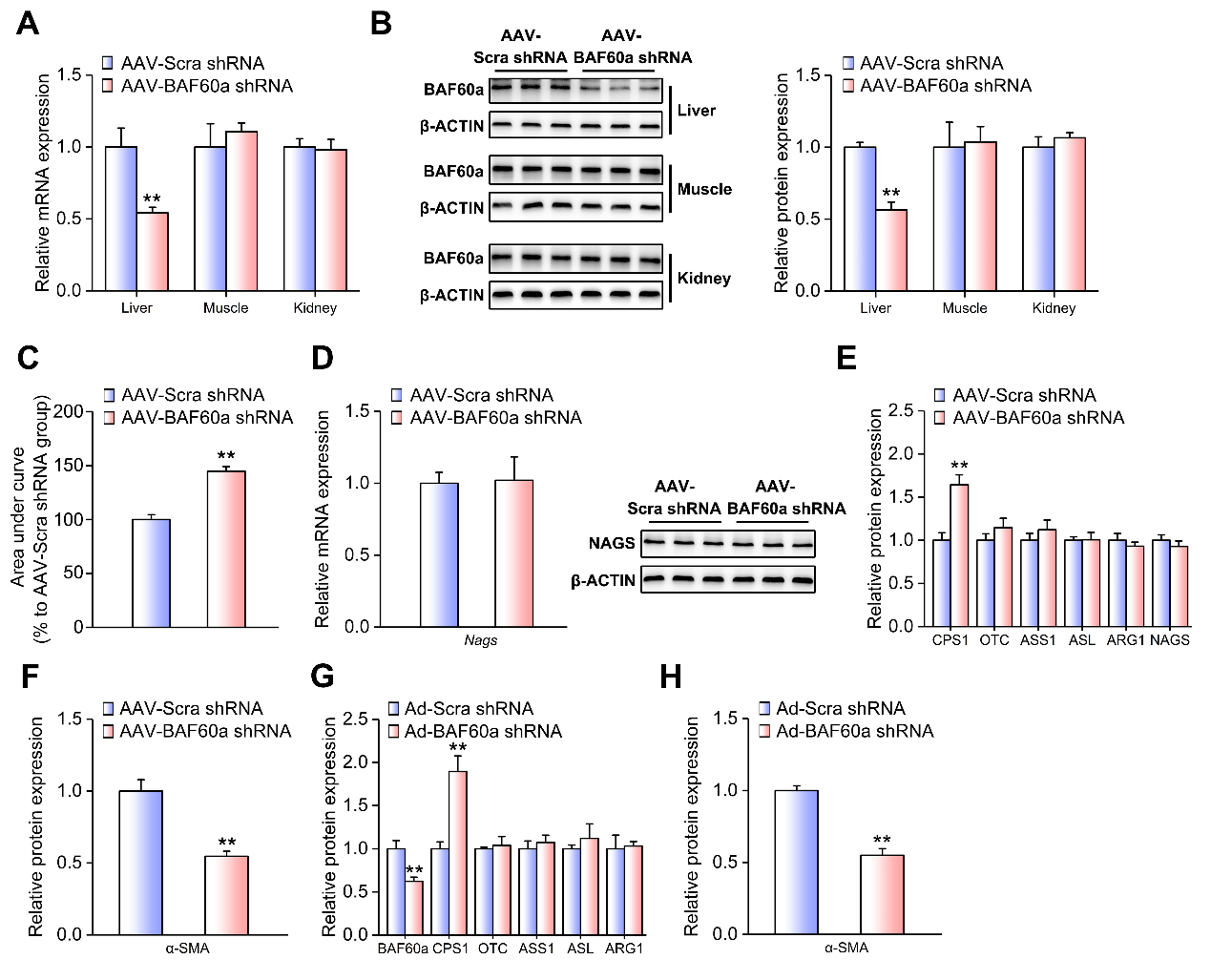


**Figure S4. Knockdown of BAF60a ameliorates HFD-induced inhibition of ureagenesis and ammonia-induced HSC activation.** (A) RT-qPCR and (B) Western blot analyses of BAF60a expression in the liver, muscle, and kidney. (C) AUC calculations for alanine tolerance tests. (D) RT-qPCR and Western blot analyses of NAGS expression. (E) Quantitative analysis of BAF60a and ureagenesis gene protein expression in Figure 4C and Figure S4D. (F) Quantitative analysis of α-SMA protein expression in Figure 4F. ^**^*P* < 0.01 vs. AAV-Scra shRNA group. n = 5. All values are presented as the mean ± SD. (G) Quantitative analysis of BAF60a and ureagenesis gene protein expression in Figure 5A. (H) Quantitative analysis of α-SMA protein expression in Figure 5D. ^**^*P* < 0.01 vs. Ad-Scra shRNA group. n = 3. All values are presented as the mean ± SD.


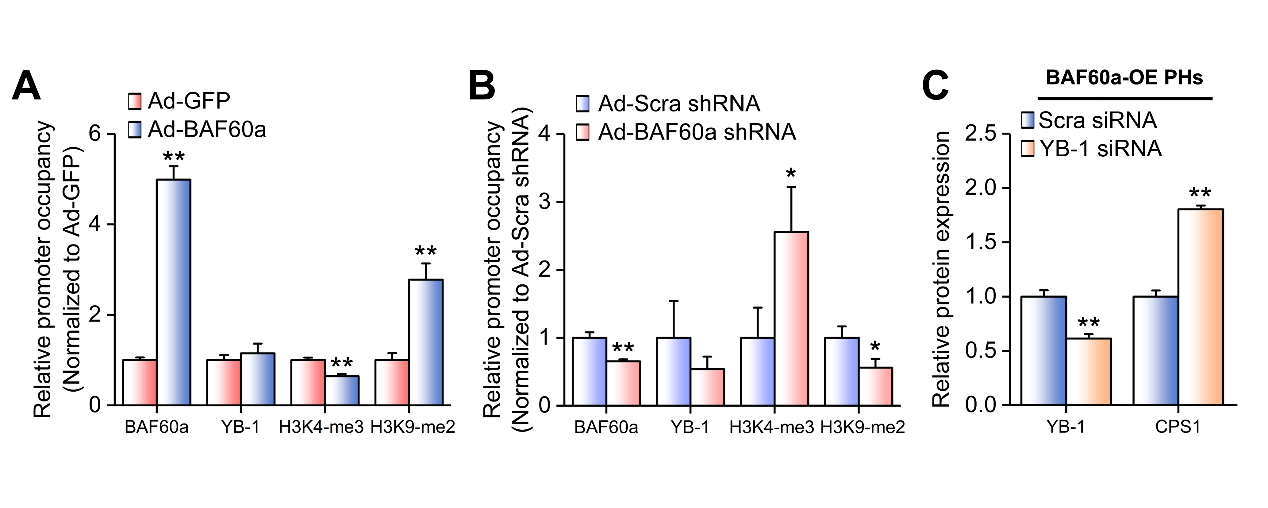


**Figure S5. BAF60a and YB-1 synergistically inhibit *Cps1* transcription.** (A) ChIP assays with indicated antibodies in mouse PHs infected by adenoviruses encoding GFP or BAF60a for 42 h, followed by 6-h DF stimulation. ^**^*P* < 0.01 vs. Ad-GFP group. n = 3. (B) ChIP assays with indicated antibodies in mouse PHs infected by adenoviruses encoding Scra shRNA or BAF60a shRNA for 24 h, followed by 24-h FFA stimulation. ^*^*P* < 0.05 and ^**^*P* < 0.01 vs. Ad-Scra shRNA group. n = 3. (C) Quantitative analysis of YB-1 and CPS1 protein expression in Figure 6D. ^**^*P* < 0.01 vs. Scra siRNA group. n = 3. All values are presented as the mean ± SD.


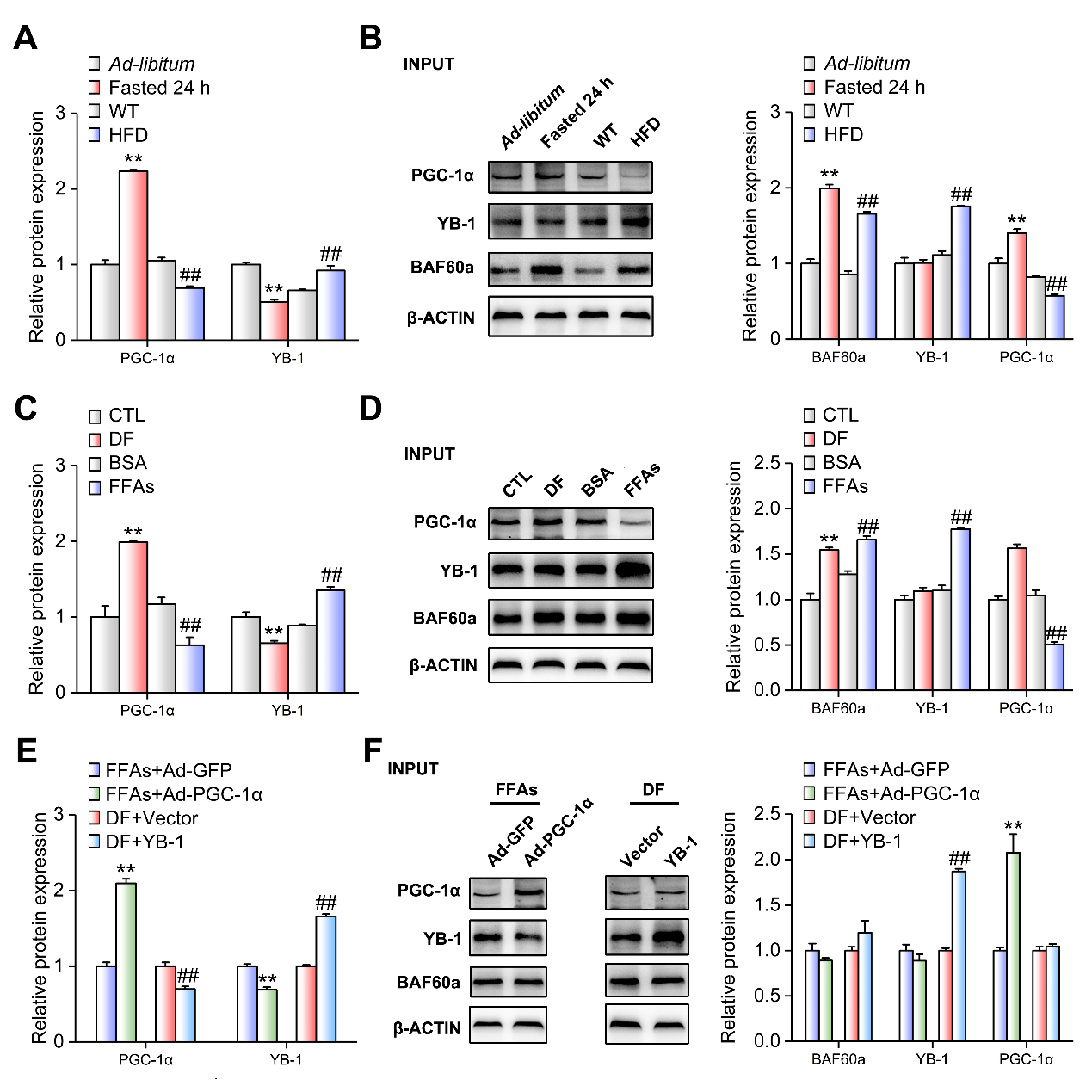


**Figure S6. BAF60a mediates crosstalk between hepatic ureagenesis and FAO pathways.** (A) Quantitative analysis of protein expression in Figure 7A. (B) Western blot analysis of PGC-1α, YB-1, and BAF60a protein expression in the liver from mice subjected to 24-h fasting or HFD feeding. ^**^*P* < 0.01 vs. ad libitum group; ^##^*P* < 0.01 vs. ND group. n = 3. (C) Quantitative analysis of protein expression in Figure 7B. (D) Western blot analysis of PGC-1α, YB-1, and BAF60a protein expression in mouse PHs treated as in Figure 7B. ^**^*P* < 0.01 vs. CTL group. n = 3; ^##^*P* < 0.01 vs. BSA group. n = 3. (E) Quantitative analysis of protein expression in Figure 7C. (F) Western blot analysis of PGC-1α, YB-1, and BAF60a protein expression in mouse PHs treated as in Figure 7C. ^**^*P* < 0.01 vs. FFAs plus Ad-GFP group; ^##^*P* < 0.01 vs. DF plus YB-1 group, n = 3. All values are presented as the mean ± SD.

**SUPPLEMENTARY TABLES**

Table S1. Sequences of shRNA and siRNA oligonucleotides for the gene knockdown.

| **shRNA oligonucleotides** | **Sequences (5’-3’)** |
| --- | --- |
| Scra shRNA | TTCTCCGAACGTGTCACGTAATTCAAGA  GATTACGTGACACGTTCGGAGAATTTTTT |
| BAF60a shRNA | GAAACTGGACCAGACTATTATTTCAAGA  GAATAATAGTCTGGTCCAGTTTCTTTTTT |
| **siRNA oligonucleotides** | **Sequences (5’-3’)** |
| Scra siRNA sense | UUCUCCGAACGUGUCACGUTT |
| Scra siRNA antisense | ACGUGACACGUUCGGAGAATT |
| YB-1 siRNA sense 1 | GGUUCCCACCUUACUACAUTT |
| YB-1 siRNA antisense 1 | AUGUAGUAAGGUGGGAACCTT |
| YB-1 siRNA sense 2 | GGUAUCGCCGAAACUUCAATT |
| YB-1 siRNA antisense 2 | UUGAAGUUUCGGCGAUACCTT |
| YB-1 siRNA sense 3 | GAACCCUAAACCACAAGAUTT |
| YB-1 siRNA antisense 3 | AUCUUGUGGUUUAGGGUUCTT |

Table S2. Lists of primer sequences for qPCR analysis

| **Mouse Genes** | **Primer Sequences (5’-3’)** |
| --- | --- |
| *36B4* Forward | GAAACTGCTGCCTCACATCCG |
| *36B4* Reverse | GCTGGCACAGTGACCTCACACG |
| *Baf60a* Forwards | GTGGAGGTGGATGACACTCTG |
| *Baf60a* Reverse | CCCACCACATCAGTCATCGT |
| *Cps1* Forward | AGTCAGCCTACAGCCTCAAC |
| *Cps1* Reverse | CCACTGTCGCTTCGTAGTCA |
| *Otc* Forward | GCTGCGTCTGACTGGACATT |
| *Otc* Reverse | TGTCAGCAGGGATACCATGAC |
| *Ass1* Forward | TCGCAGACAGGTGGAGATTG |
| *Ass1* Reverse | GGTGACAGGGATGGGGATTC |
| *Asl* Forward | GGAGCTACTCCGTGCAGAAC |
| *Asl* Reverse | GTTTTTCTTCTGGGGCATCA |
| *Arg1* Forward | CAGCACTGAGGAAAGCTGGT |
| *Arg1* Reverse | CAGACCGTGGGTTCTTCACA |
| *Baf60b* Forward | GGATCTGGAGTATCCCTGGC |
| *Baf60b* Reverse | CGGGTTCCGGGATCTTCTAC |
| *Baf60c* Forward | AACCAGCAGGAGATCAGTGC |
| *Baf60c* Reverse | CTGCGCTGCTGGATCTTACA |
| *Nags* Forward | CACGGAAGTGAAGCCAGCTA |
| *Nags* Reverse | ACATTGAAGGCCGTCTGGTT |
| *Ybx1* Forward | AAGTGATGGAGGGTGCTGAC |
| *Ybx1* Reverse | TGACCTTGGGTCTCATCTCC |
| *α-Sma* Forward | CCCTGGAGAAGAGCTACGAAC |
| *α-Sma* Reverse | TACCCCCTGACAGGACGTTG |
| *Tgf-β* Forward | AGGGCTACCATGCCAACTTC |
| *Tgf-β* Reverse | CCACGTAGTAGACGATGGGC |
| *Col1a1* Forward | AGCACGTCTGGTTTGGAGAG |
| *Col1a1* Reverse | GACATTAGGCGCAGGAAGGT |
| *Cps1* ChIP Forward | TGCATTATTAGCAAGGTACTGCCC |
| *Cps1* ChIP Reverse | TTCCTTAGCCCCTCCTCCCAAGCTG |
| *Acaa1b* ChIP Forward | GCACTGATGAGGGCATCTC |
| *Acaa1b* ChIP Reverse | CTAAGCTGGATGCTTGAGTAC |
